# Herpesviruses dUTPases: A New Family of Pathogen-Associated Molecular Pattern (PAMP) Proteins with Implications for Human Disease

**DOI:** 10.3390/pathogens6010002

**Published:** 2016-12-28

**Authors:** Marshall V. Williams, Brandon Cox, Maria Eugenia Ariza

**Affiliations:** 1 Department of Cancer Biology and Genetics, Wexner Medical Center, Ohio State University, Columbus, OH 43210, USA; Williams.70@osu.edu; 2 Institute for Behavioral Medicine Research, Ohio State University, Columbus, OH 43210, USA; Brandon.Cox@osumc.edu

**Keywords:** herpesviruses, Epstein–Barr virus, deoxyuridine triphosphate nucleotidohydrolase, Toll-like receptor 2, myalgic encephalomyelitis/chronic fatigue syndrome, autoimmune diseases, lupus nephritis, cancer

## Abstract

The human herpesviruses are ubiquitous viruses and have a prevalence of over 90% in the adult population. Following a primary infection they establish latency and can be reactivated over a person’s lifetime. While it is well accepted that human herpesviruses are implicated in numerous diseases ranging from dermatological and autoimmune disease to cancer, the role of lytic proteins in the pathophysiology of herpesvirus-associated diseases remains largely understudies. Only recently have we begun to appreciate the importance of lytic proteins produced during reactivation of the virus, in particular the deoxyuridine triphosphate nucleotidohydrolases (dUTPase), as key modulators of the host innate and adaptive immune responses. In this review, we provide evidence from animal and human studies of the Epstein–Barr virus as a prototype, supporting the notion that herpesviruses dUTPases are a family of proteins with unique immunoregulatory functions that can alter the inflammatory microenvironment and thus exacerbate the immune pathology of herpesvirus-related diseases including myalgic encephalomyelitis/chronic fatigue syndrome, autoimmune diseases, and cancer.

## 1. Introduction

The human herpesvirus family consists of eight members and is divided into three subfamilies, α, β, and γ, based on their cellular tropism and genomic structure. These viruses are ubiquitous in nature and, following a primary infection, establish latency and can be reactivated over a person’s lifetime. The human herpesviruses are implicated in numerous diseases ranging from dermatological and autoimmune diseases to cancer. Most of these studies have focused on the cellular damage caused during replication of the virus, as well as the mechanisms by which herpesviruses evade the host immune response. However, few studies have asked the question of whether a protein that is expressed during abortive or productive virus replication, namely a deoxyuridine triphosphate nucleotidohydrolase (dUTPase), can contribute to the pathophysiological alterations that occur in diseases associated with human herpesviruses infections.

The herpesviruses encode for several genes that are homologous among the various members, including a dUTPase. dUTPases represent a family of metalloenzymes that catalyze the hydrolysis of dUTP to dUMP and pyrophosphate [1], thus preventing the incorporation of dUTP into DNA by DNA polymerases. dUTPases are divided into three subgroups based on their structure and specificity for dUTP. The homotrimeric subgroup, which is found in most organisms including some retroviruses and poxviruses, is the largest and these dUTPases exhibit high specificity for dUTP. The homotrimeric dUTPases are composed of three identical polypeptides. Each polypeptide contains five conserved amino acid motifs that contribute to the formation of the catalytic site and there are three catalytic sites in the holoenzyme.

The monomeric dUTPases, which are thought to have arisen from the trimeric dUTPases by gene duplication [2], are found exclusively in herpesviruses [3]. Structural data of the Epstein–Barr virus (EBV) dUTPase demonstrate that the single catalytic site mimics the catalytic site of homotrimeric dUTPases in that it is comprised of five highly conserved motifs [4]. Sequencing analyses have demonstrated the presence of an additional conserved motif (motif 6) of unknown function in the herpesviruses’ dUTPases that is absent in the homotrimeric dUTPases [5]. Although the human β herpesviruses (human cytomegalovirus, HCMV; human herpesvirus 6A and B, HHV-6; human herpesvirus 7, HHV-7) contain putative genes encoding a dUTPase, studies with HCMV (UL72) [6] and HHV-6A (U45) [7] have shown that these genes do not encode for a protein with functional dUTPase activity. This is not surprising since examination of the protein expressed by these viruses revealed that they lack the five conserved motifs typical of homotrimeric and monomeric dUTPases. However, they do contain motif 6 characteristic of α and γ human herpesviruses dUTPases.

**Figure 1:**
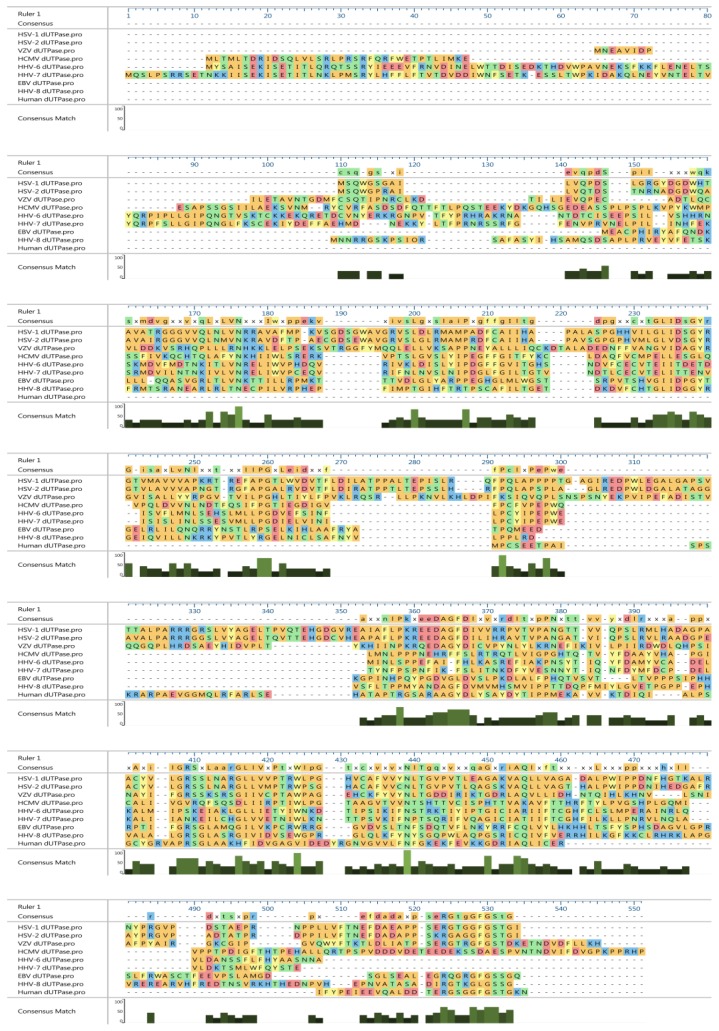
Clustal alignment of human herpesviruses and human dUTPases using DNASTART MegaAlignPro software. Amino acids (aa) are colored according to their side chain, red: acidic; blue: basic; green: neutral; orange: non-polar; and yellow: aromatic. The Consensus Match histogram shows the percentage of matches to the consensus at each position in the active block. The Consensus Match histogram is calculated by dividing the total score for the called consensus character by the number of sequences at the position. As agreement increases, the bar height increases and appears in a lighter shade of green. Herpes simplex virus type 1 (HSV-1; UL50): 371 aa; Herpes simplex virus type 2 (HSV-2; UL50): 369 aa; Varicella–Zoster virus (VZV; ORF8): 396 aa; Human cytomegalovirus (HCMV; UL72): 388 aa; Human herpesvirus-6 (HHV-6: U45): 376 aa ; Human herpesvirus-7 (HHV-7; U45): 379 aa; Epstein–Barr Virus (EBV; BLLF3): 278 aa; Human herpesvirus 8 (HHV-8; ORF54): 317 aa; human nuclear dUTPase isoform: 164 aa.

The last subgroup is the homodimeric dUTPases, which were identified in *Leshmania major* [8,9], *Trypansoma cruzi* [10], *Caenorhabditis elegans* [11], and *Campylobacter jejuni* [12]. The homodimeric dUTPases differ not only structurally from the monomeric and homotrimeric dUTPases, but they also exhibit broader substrate specificity. Furthermore, sequence comparisons have demonstrated that the dimeric dUTPases lack the five consensus amino acid motifs found in mono- and trimeric dUTPases and they are evolutionary related to the dCTPase-dUTPase of bacteriophages T2 and T4 [9]. Protein sequence alignments of the herpesviruses and human nuclear dUTPases are shown in Figure 1.

Studies regarding the role(s) of virus-encoded dUTPases on viral replication processes have focused primarily on the enzymatic activity of the protein. It is generally assumed that the primary function of virus-encoded dUTPases is to maintain a low dUTP pool during viral replication and thus prevent dUTP incorporation into the replicating viral genome. However, critical studies to demonstrate this premise as well as studies to examine alterations in dUTP metabolism following virus infections have not been performed. Scientific literature reports concerning HSV-1 and VZV dUTPases have shown that neither protein is required for viral replication in vitro [13,14]. However, this may reflect the ability of the host dUTPase to compensate for the virus dUTPase under these conditions [15,16,17]. Pyles et al. [18] reported that HSV-1 dUTPase (UL50) defective mutants were attenuated for neurovirulence, neuroinvasiveness, and reactivation from latency in vivo. Likewise, Song et al. [19] demonstrated that the dUTPase gene (ORF54) of murine γ-herpesvirus-68, a surrogate model for human γ-herpesviruses, was required for efficient replication in the lungs of infected animals. Surprisingly, few studies have asked the question of whether the viral dUTPases may have additional functions involved in pathogenesis. A summary of herpesviruses dUTPase properties and recent findings concerning their immune modulatory functions is given in Table 1.

Our group has performed pioneering studies that have led to the establishment of significant groundwork concerning the potential role of some dUTPases in the pathophysiology of diseases associated with viral infections. The objective of this review is to provide a summary of these findings using the EBV-dUTPase as a prototype.

The EBV-dUTPase is encoded by the BLLF3 gene and is expressed as an early gene product during productive replication of EBV. There is increasing evidence supporting the premise that EBV as well as other human herpesviruses also undergo abortive lytic replication and that viral-encoded proteins may be released from infected cells through pyroptosis, a highly inflammatory form of programmed cell death [35]. Although human and EBV dUTPases lack consensus secretory signal sequences, several studies have reported that the nuclear isoform of the human dUTPase is released from stressed cells [36,37]. A later study by Buschow et al. [38] demonstrated that the human dUTPase protein was secreted in membrane vesicles/exosomes from B cells. Interestingly, the human adenovirus type 9 E4-ORF1 encodes for an ancestral dUTPase [39] that is also targeted to membrane vesicles [40]. In line with these studies, we have demonstrated that the EBV-dUTPase is released in exosomes from B cells during abortive lytic replication of the virus [29]. Because exosomes are important in cell–cell communication, these vesicles may provide a route of transmission of EBV-dUTPase to other cells and/or sites of the body, enabling the viral dUTPase to modulate the cellular microenvironment by acting as an intercellular signaling molecule. Thus, dUTPase-containing exosomes provide a possible mechanism by which the EBV-dUTPase may contribute to the pathophysiology of EBV-related diseases [29].

## 2. Herpesviruses’ dUTPases and Innate Immunity

The innate immune response is an early line of defense, which plays a key role in the protection of a host from invading pathogens including viruses. Viruses, as well as other pathogens, encode for various proteins containing pathogen associated molecular patterns (PAMPs) that are recognized by immune-sensor molecules referred to as pattern recognition receptors (PRRs). Toll-like receptors (TLR) are PRRs and are responsible for the primary recognition of a broad range of pathogens [41], leading to the initiation of innate and adaptive immune responses [42,43,44].

TLR2 is localized on the cell surface and forms homodimers or heterodimer complexes with either TLR1 or TLR6. Activation of TLR2 has been implicated in oncogenesis [45], autoimmune diseases (AD) including multiple sclerosis (MS), rheumatoid arthritis (RA), systemic lupus erythematosus (SLE), Sjogren’s syndrome (SS), and Systemic sclerosis (SSc) [46], as well as in neurological dysfunction/disorders [47]. TLR2 is vital for the initiation of the antiviral defense and requires the recognition of structural components (viral PAMPs) in a large number of viruses including hepatitis C virus [48], measles virus [49], and human immunodeficiency virus type 1 (HIV-1) [50]. In the case of members of the *Herpesviridae* family, TLR2 is important for the recognition of herpes simplex type 1 (HSV-1) [51,52], VZV [53], HCMV [54,55,56], and EBV [57]. Once engaged, TLR2 triggers a signaling cascade that results in the activation of various transcription factors and pro-inflammatory mediators, which contribute to the development and progression of disease.

We have demonstrated that the EBV-dUTPase triggers NF-κB activation through engagement of TLR2 homodimers, while the dUTPases expressed by HSV-2, HHV-6A, HHV-8, and VZV require ligation of the TLR2/1 heterodimer complex to activate NF-κB [7,27]. We have also demonstrated that these viral dUTPases are capable of differentially inducing the secretion of the pro-inflammatory T_H_1/T_H_17 cytokines IL-1β, IL-6, IL-8, IL-12p70, TNF-α, and IFN-γ as well as the anti-inflammatory cytokine IL-10 (*p* < 0.001) in human primary immune cells [7,25,26,27]. These differences in the ability of the herpesviruses dUTPases to induce NF-κB activation and cytokine secretion may reflect differences in intrinsic properties of each viral protein including binding affinities of the dUTPases to TLR2 or TLR2/TLR1 and the overall structure of the dUTPases. The size and sequence homologies vary greatly; the EBV dUTPase is the smallest (278 amino acids) while the VZV dUTPase is the largest (396 amino acids). The greatest identity (31%) occurs between the EBV and HHV-8 dUTPases and the least identity (21%) occurs between the EBV and HHV-6 A and B dUTPases.

## 3. Stress and Herpesvirus Reactivation

Physical and psychological stresses have been shown to have direct effects on biological processes and/or behavioral patterns that influence disease risk for developing autoimmune diseases, myalgic encephalomyelitis/chronic fatigue syndrome (ME/CFS) [58,59,60,61], coronary artery disease [26,28], and cancer [61,62,63]. These stressors can in fact impact the virome both directly and indirectly.

Herpesviruses are ubiquitous viruses; in particular, EBV, HCMV, HHV-6, HHV-7, and VZV have a prevalence of greater than 90% in all adult populations. Infections with these herpesviruses occur early in life and, with the exception of VZV, which is the etiological agent of chickenpox and shingles, most primary infections are asymptomatic [64,65,66,67,68,69]. It is well established that physical and psychological stresses induce the reactivation of herpesviruses [61,70,71,72,73] and it is thought to occur as a result of interactions between the central nervous system, the endocrine system, and the immune system. Although these studies have suggested that viral reactivation is due in part to an impaired immune system, these complex interactions must also reflect changes in virus gene expression, which allow for a switch from latent genes to lytic/abortive-lytic gene expression. Studies by Yang et al. [74] have shown that glucocorticoids activate EBV lytic replication in vitro by upregulating the expression of the immediate early BZLF1 gene. Conversely, Cliffe et al. [75] reported that a neuronal pathway involving activation of c-Jun N-terminal kinase (JNK), common to many stress responses, is essential for initial HSV gene expression during reactivation. These results, while limited, demonstrate that components of the stress response might be essential for causing the switch from latency to reactivation.

Reactivation of the herpesviruses following stress has focused primarily on the dysregulation of the immune system. One area that has been overlooked, though, is whether virus proteins produced during lytic/abortive-lytic replication contribute to the decreased immune homeostasis following stress. In most cases, reactivation of these viruses may not result in productive infections but rather abortive infections, especially in the case of EBV [76,77,78]. However, even if productive replication occurs it may not result in clinical symptoms. Thus, the potential contributions of the virus to immune homeostasis in the host are “hidden”.

## 4. Herpesviruses dUTPases and Human Disease

### 4.1. Myalgic Encephalomyelitis/Chronic Fatigue Syndrome

Myalgic encephalomyelitis/chronic fatigue syndrome (ME/CFS) is a chronic multisystem illness of unconfirmed etiology [79]. While the onset of greater than 50% of ME/CFS cases is associated with acute “flu-like” symptoms [80], the data concerning a causal relationship between a virus and ME/CFS has not been conclusively demonstrated and remains a challenge. Conflicting results regarding the potential role of viruses in ME/CFS may be due in part to the heterogeneity of the patient populations, and in part to multiple case definitions. Perhaps the most significant problem that has led to contradictory data came from the type of approaches/assay endpoints used to examine the relationship between viruses and ME/CFS, and the interpretation of the data. These approaches consisted primarily of serological studies using many different virus proteins as antigens; some that are expressed early during the replication cycle of the virus and others that are expressed late. Since the antibody pattern against a virus early protein would not be the same as the antibody pattern against a virus late protein, serology studies using different virus proteins (early vs. late) as antigens could lead to variable results [81,82,83,84,85,86,87,88,89,90,91]. Other studies have employed culture methods and polymerase chain reaction (PCR) methods to determine increased viral load when compared to controls [64,81]. This approach is complicated in the case of the herpesviruses since most adults are latently infected with these viruses and spontaneous asymptomatic reactivation occurs periodically during a person’s lifetime. Furthermore, PCR and culturing approaches will not demonstrate abortive lytic replication, which is reported to occur with several herpesviruses [76,77,78,92,93]. Surprisingly, none of these studies have approached the possibility that two or more herpesviruses may act synergistically and that virus-encoded proteins, rather than the viruses themselves, may act as drivers of or contribute to the pathophysiological alterations observed in a subset of patients with ME/CFS.

A major problem associated with studies concerning CFS is that while patients exhibit similar symptomology, the triggers and thus the pathways associated with the development of these symptoms may be different. Furthermore, no animal models have been developed that mimic CFS. Our studies have demonstrated that there are elevated antibody levels to the EBV-dUTPase in a subset of ME/CFS patients. Recently, we extended these studies to include ME/CFS patients from a “good day bad day study” and of the 74 patients examined (four longitudinal samples from each patient) 32.34% (*n* = 24) were negative for antibodies to HHV-6, EBV-, and the human nuclear encoded dUTPase proteins. Some patients expressed antibodies to only HHV-6 (2.7%), EBV (5.41%), or human (6.76%) dUTPases, some co-expressed antibodies (20.27%) to the HHV-6 and EBV dUTPases, but the majority (28.38%) co-expressed antibodies to the HHV-6, EBV-, and human dUTPases [94]. These results not only suggest that there is reactivation of multiple herpesviruses in a subset of patients with ME/CFS, but also that the dUTPases are produced physiologically at sufficient levels to elicit a humoral response. More importantly, as we have shown previously, these viral dUTPases can induce the secretion of pro-inflammatory T_H_1/T_H_17 cytokines known to be increased in some ME/CFS patients. Thus, the presence of physiological levels of multiple viral dUTPases may promote/enhance the immune dysregulation observed in some ME/CFS patients. Focusing on the EBV-dUTPase, we have also demonstrated using a mouse model that the EBV- dUTPase induced sickness and anxiety-like behaviors, impaired learning, and memory responses and that chronic restraint stress exacerbated these symptoms [30,33,95].

A common finding in some patients with ME/CFS is a reduction in NK cell numbers and function. While HHV-8 is not associated with ME/CFS, Madrid and Ganem [34] reported that the dUTPase encoded by the ORF54 gene of HHV-8 downregulated, independent of its enzymatic activity, NKp44L, an uncharacterized ligand for the NK cell activating receptor NKp44. In addition, the ORF54 protein downregulated the expression of specific cytokine receptors, including IL-23R and IFNAR1, suggesting that the HHV-8 dUTPase may alter membrane protein trafficking. Similar results were not observed with EBV or HSV dUTPases. Interestingly, Schmiedel et al. [96] recently reported that HHV-6B downregulates NK cell activation and, although they did not identify the viral protein(s) responsible for this effect, their results suggest it is an early protein. Conversely, our in vitro studies using human primary NK cells demonstrated that direct treatment of NK cells with the EBV-dUTPase did not have an effect on the ability of NK cells to kill K-562 target cells but it did synergize with IL-2 to strongly stimulate the production of IFN-γ (unpublished data).

There are multiple reports in the literature suggesting a role for HHV-6 [81,82,83,84,85,86] and EBV [87,88,89,90,91] in ME/CFS, but none have examined whether multiple herpesviruses may be reactivated simultaneously in patients, thus contributing to the pathophysiology. The hypothesis that reactivation of multiple herpesviruses may be involved with ME/CFS is supported by clinical studies demonstrating improvement of symptomology in a subset of patients following long-term therapy with valganciclovir, a potent inhibitor of herpesvirus replication, and relapse upon discontinuation of treatment [97,98,99,100]. Further support for a possible involvement of EBV in a subset of patients with ME/CFS comes from studies that reported improvement of symptoms following single treatment and maintenance therapy with Rituximab, an anti-CD20 antibody that effectively depletes B cells but not plasma cells [101,102,103]. According to the investigators, the two- to eight-month delay in symptoms following initiation of therapy suggested that the time delay was needed to eliminate circulating autoantibodies that naturally precedes the observed improvements in ME/CFS [103]. Another possibility relates to the biology of EBV. EBV remains latent in a pool of memory B cells. Differentiation of memory B cells to plasma cells results in the reactivation of the latent virus, primarily abortive lytic replication [76,77,78]. Thus, the delay in clinical improvement may reflect the time that it takes to deplete the memory B cell pool.

Altogether these data suggest that the expression of these herpesvirus dUTPases may contribute to the symptomology observed in a subset of ME/CFS patients. Furthermore, our data suggest that not only could anti-HHV-6 and EBV-dUTPase antibodies be useful as potential biomarkers for ME/CFS, but also the interaction of these dUTPases with TLR2 or TLR2/TLR1 could be a novel target for the development of therapeutic agents.

### 4.2. Autoimmune Diseases

Autoimmune diseases (AD) are complex diseases that develop as a consequence of dysregulation of the immune system, resulting in loss of self-tolerance and ultimately tissue damage and/or disruption of physiological processes. Genetic and environmental factors are known to contribute to AD. Various environmental factors, including some viruses, have been proposed to function as triggers of AD. However, the mechanisms by which these viruses may promote the loss of tolerance or enhance the development of AD in genetically susceptible individuals are unknown. Literature reports point to several herpesviruses as possible environmental triggers for AD including HCMV, which has been linked to SLE, SSc, diabetes mellitus type 1, and RA [104]. Also, HHV-6 has been linked to multiple sclerosis (MS), autoimmune connective tissue disease, and Hashimoto’s thyroiditis [105], while EBV has been associated with SLE, MS, RA, and SS [106]. Linkage of these viruses to AD is based primarily on sero-epidemiological studies, demonstration of cross-reactivity of antibodies between cellular and viral proteins (molecular mimicry), and, in some cases, increases in viral load. However, it is possible that the virus-specific immune responses observed in AD patients may reflect the sensitivity of the viruses to perturbations in the immune system allowing for reactivation of latent virus and, thus, the virus-mediated effects may be a consequence of AD rather than a cause [107]. While most studies have focused on herpesviruses as triggers for AD, none have asked the question of whether proteins expressed by these viruses could be driving or contributing to the pathophysiology of the disease. In this regard, we recently performed animal and human studies to determine the role of EBV-dUTPase in lupus nephritis (LN) pathophysiology.

LN is the most common solid organ manifestation observed in SLE patients and poor renal function is a predictor of overall survival. The biochemical and cellular processes leading to LN involve alterations in clearance of dead cells, activation of antiviral immunity pathways, and aberrant lymphocyte proliferation [108,109,110]. It is well established that EBV is an environmental risk factor with strong links to SLE pathogenesis. However, the mechanism by which EBV may contribute to SLE-LN is not known.

There is increasing evidence from murine models and human LN studies [111,112,113,114,115,116,117] that TLR2 and the pro-inflammatory cytokine IL-17 may play a role in the pathophysiology of LN. In support of this premise, recent studies have demonstrated that the TLR2/MyD88/miRNA155/Ets-1 pathway is required for the production of autoantibodies that form DNA-containing immune complexes [112] and that increased TLR2 expression promotes IL-17 production in SLE patients [117]. Furthermore, it has been shown that IL-17 serum levels were significantly increased in patients with SLE and positively correlated with SLE Disease Activity Index (SLEDAI) scores [118]. In line with these findings, extensive studies by our group have demonstrated that the EBV dUTPase activates TLR2 in human immune cells [7,27,29] and induces the production of pro-inflammatory T_H_1/T_H_17 cytokines [7,27,29,30], which have been implicated in the pathogenesis of SLE [113,114].

Using the NZM2410/J SLE mouse model, we also demonstrated that intramuscular administration of the EBV-dUTPase protein significantly enhanced glomerulonephritis characterized by interstitial/tubular cellular infiltrates, increased IgG complex formation and C3 deposition in glomeruli as well as a strong induction of IL-17 in glomeruli and tubules [32].

Collectively, our study indicates that it is unlikely that EBV acts as a trigger for SLE or LN. However, it does provide important evidence supporting a role for EBV-dUTPase in the exacerbation of the immune pathology associated with LN.

**Figure 2:**
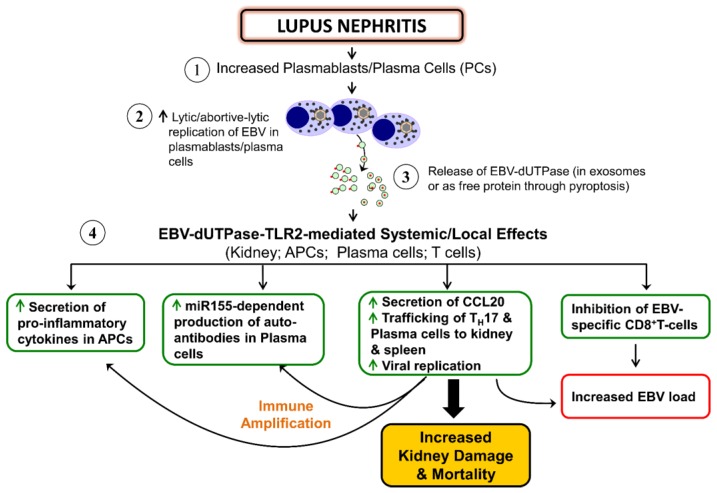
Proposed mechanism(s) by which EBV reactivation enhances LN pathology. We hypothesize, based on our studies [7,25,26,27,28,29,32], that the increased differentiation of autoimmune B cells into plasmablasts/plasma cells observed in lupus nephritis patients with active disease induces the reactivation of EBV. This results in the increased expression of EBV-dUTPase and its release from target cells in exosomes and/or through cell lysis in a process known as pyroptosis. The EBV dUTPase contributes to the continuous immune activation observed in a subset of LN patients by activating TLR2 signaling, driving the differentiation of naïve CD4^+^ T cells into T_H_17, enhancing the development of autoantibodies and impairing EBV-specific CD8^+^ T cell function, which leads to decreasing immune surveillance to EBV and increased viral load, ultimately causing kidney and/or target organ damage.

While EBV has been implicated in several autoimmune diseases including SLE, RA, MS, and SS, the mechanism by which the virus contributes to these diseases remain unknown. However, when one examines the biology of the virus as it relates to these diseases it is quite possible that a common mechanism exists. Each disease is characterized by the infiltration of inflammatory cells including plasmablasts and plasma cells. The differentiation of memory B cells into plasmablasts/plasma cells results in abortive lytic replication of EBV and expression of the dUTPase, which may be released from infected cells through pyroptosis and/or in exosomes. Moreover, exosomes have been implicated in the pathogenesis of RA and SLE [119]. EBV-dUTPase stimulation of human dendritic cells and macrophages in the microenvironment would result in the increased expression and secretion of T_H_1/T_H_17 proinflammatory cytokines (TNF-α, IL-1β, IL-6, IL-8) as well as the anti-inflammatory cytokine IL-10 [7,25,27,29]. The production of IL-17 by T_H_17 cells promotes chronic inflammation, which is enhanced by IL-8-mediated recruitment of neutrophils and macrophages to the site of inflammation. This, coupled with a T_H_17/regulatory T (Treg) cell imbalance, could result in a state of chronic inflammation that might be exacerbated by the viral dUTPase [32]. A schematic diagram summarizing our findings and the proposed mechanism by which EBV-dUTPase contributes to the pathophysiology of lupus nephritis and potentially other AD is shown below (Figure 2).

### 4.3. Carcinogenesis

EBV and HHV-8 are γ-herpesviruses associated with several human malignancies. EBV has been associated with several B cell malignancies, including AIDS/post-transplantation lymphoma, Burkitt’s lymphoma, Hodgkin’s lymphoma, non-Hodgkin’s lymphoma, as well as epithelial cell malignancies such as nasopharyngeal carcinoma (NPC) and some gastric carcinomas. Conversely, HHV-8 has been associated with Kaposi sarcoma (KS), an endothelial cell malignancy; the B-cell malignancies primary effusion lymphoma (PEL) and the plasma cell variant of Multicentric Castleman’s Disease (MCD).

While cells infected with EBV in EBV-associated “tumors” are generally expressing one type of the latency programs, a small number of cells in these tumors express EBV genes associated with lytic replication of the virus [120,121,122,123,124,125,126,127], suggesting that products from lytic or abortive-lytic replication of EBV may contribute to tumor growth/survival. This premise is supported by data from in vitro studies as well as studies using SCID and humanized mouse models [128,129,130,131]. Similar results have also been reported for Burkitt’s lymphoma [132]. Likewise, HHV-8 associated tumors exhibit low levels of virus reactivation and epidemiological studies support the premise that lytic/abortive-lytic replication is important in the initiation and progression of these tumors [133,134]. Thus, virus proteins expressed under these conditions could be regarded as potential targets for treatment. However, the potential role of these lytic replication-associated proteins in immune evasion in immunocompetent or immunosuppressed individuals as well as in promoting tumor growth/survival of malignant cells is unknown.

Numerous studies have demonstrated the importance of the tumor microenvironment in tumor growth and disease progression. Within this environment, stromal cells, immune cells, and vascular cells “cross-talk” with tumor cells. A question that comes to mind is could the dUTPases from the oncogenic γ-herpesviruses EBV and HHV-8 contribute to the tumor microenvironment? Using microarray, proteome array, and functional studies we have demonstrated [7,25,26,27,29] that the EBV dUTPase: (i) induces the expression and secretion of IL-6, a known modulator of B cell differentiation [135] and key driver of plasmablast proliferation and survival [136]; (ii) induces the expression of IL-10, a tolerogenic, anti-inflammatory, and pleiotropic cytokine [137], which is capable of inhibiting CD8^+^ T cell function in viral infections [138]; (iii) upregulates the expression of BIC/microRNA-155 an oncomicroRNA associated with aberrant inflammatory responses, enhanced B cell transformation, and the development of Tregs [139,140]; and, finally, (iv) upregulates the expression of CCL20 (335-fold), a strong inducer of Treg migration/trafficking into the tumor environment thus, dampening the immune response to EBV [141]. Interestingly, effector and memory T cells express TLR2, which can act as a T cell co-stimulatory molecule [142], and signaling through TLR2 is reported to alter the proliferation and function of CD4^+^CD25^+^ Tregs [143,144,145,146]. These results suggest that the EBV-dUTPase may alter the tumor microenvironment by impairing the function, proliferation, and/or migration of Tregs.

It is established that cytotoxic T-lymphocytes (CTLs) are responsible for limiting the proliferation and for clearing cells latently infected with EBV [147]. However, our knowledge concerning whether viral early proteins, in particular the EBV-dUTPase, may modulate T-cell responses to cells productively or latently infected with EBV is rather limited [148]. Using an in vitro model of EBV superinfection [149], we demonstrated that the proliferation/expansion of EBV-transformed B cells was enhanced by the EBV-dUTPase relative to untreated cells infected with EBV [150]. This finding, together with a previous study by our group demonstrating that the EBV-dUTPase inhibited T cell blastogenesis [25], suggests that the EBV-dUTPase may be preventing the killing of B cells latently infected with EBV by either inducing the proliferation/survival of B cells and/or impairing the function of EBV-specific CTLs.

While the mechanism(s) by which the EBV-dUTPase may inhibit CD8^+^ T cell function is not known, our microarray analysis of EBV-dUTPase in human dendritic cells revealed that the dUTPase modulates a select group of genes involved in the regulation of T-cell function, including a gene encoding for a secreted L-phenylalanine oxidase, in mature dendritic cells, that downregulates the expression of TCR∂ chain, thus inhibiting T-cell proliferation; PD-L2, which binds to the inhibitory receptor PD-1 on T-cells; and the inducible costimulatory (ICOS) ligand ICOSL [29,150]. Binding of ICOSL to ICOS on activated T-cells results in increased IL-10 expression and recent data suggest that this interaction may be involved with the expansion of Foxp3^+^ Treg cells [151,152].

Altogether, these data suggest that the expansion of plasmablasts/plasma cells within the tumor microenvironment [153,154,155,156] may result in the increased release of EBV-dUTPase from these cells due to lytic/abortive-lytic replication of EBV [76,77,78]. Ligation of the EBV-dUTPase with TLR2 on CD14^+^ APCs, and T-cells results in the activation of specific pathways and the production of polarizing cytokines (IL-6 and IL-10) towards an environment that favors the proliferation of B cells while simultaneously diminishing the CTL response against latently EBV-infected B cells and also increasing the formation of a suppressive Treg population, ultimately leading to the proliferation/survival of tumor cells.

## 5. Concluding Remarks

The dUTPases expressed by the human herpesviruses constitute a new class of PAMP proteins that possess novel immunomodulatory functions independent of their enzymatic activity. Ligation of TLR2 or TLR2/1 by the herpesvirus dUTPases leads to the activation of NF-κB and subsequent modulation of downstream genes involved in innate and adaptive immunity, chronic inflammation, and oncogenesis. Studies using the EBV-dUTPase as a prototype of the monomeric herpesviruses dUTPases have demonstrated that the protein acts as an intercellular signaling molecule capable of altering the cellular microenvironment and, thus, it may be important in the pathophysiology of EBV-related diseases. For instance, trafficking of plasma cells to sites of inflammation could result in EBV reactivation and the production of EBV-dUTPase, which contributes directly to immune pathology, as demonstrated by our studies on LN. Such immune pathology would be enhanced under conditions of chronic inflammation. Genetic predisposition, stress, and environmental insults that alter the immune response to these viruses could result in viral reactivation and the production of the dUTPase. Considering that herpesviruses exhibit different cellular tropisms and most humans are latently infected with several members of this family, simultaneous reactivation of multiple herpesviruses could occur and thus “cooperate” to further enhance immune dysfunction and alter the cellular microenvironment to promote oncogenesis as well as the development of autoimmune diseases in genetically predisposed individuals.

There is accumulating evidence indicating virus cooperation either directly through viral gene products or indirectly through perturbations in the immune system or the environment. Reactivation of multiple herpesviruses has been reported in transplant recipients [157,158,159], patients with sepsis [160] and drug-induced hypersensitivity syndrome (DISS), also known as drug rash, or those with eosinophilia and systemic symptomology (DRESS) [161,162,163,164,165,166]. The temporal order in which these viruses are reactivated and whether they interact to contribute to symptomology remains unknown. However, it has been suggested that a complex interplay among several herpesviruses including HHV-6 and EBV is responsible for the symptomology observed in DISS/DRESS patients [161,162,163,164,165,166].

Previous studies have also shown that HHV-6 replication may result in the reactivation of EBV [167] and human immunodeficiency virus type 1 (HIV-1) [168] in vitro. In line with these studies, we have recently demonstrated that the EBV-dUTPase transactivates the LTR of HIV-1 in vitro [150]. Potential interactions between the herpesviruses dUTPases are not limited to interactions among themselves but also to other members of the virome, including HIV-1 [169] and human papillomavirus [170], as well as members of the microbiome to promote chronic periodontitis [171,172] and malaria [173,174]. Additional studies are required to address these possibilities.

dUTPases are also encoded by other human viruses, including human endogenous retrovirus-K (HERV-K) and vaccinia virus. Furthermore, ancestral forms of dUTPases have been reported in human adenovirus and HIV-1. While these viral dUTPases are homotrimeric proteins, we have shown that the HERV-K dUTPase protein also functions as a TLR2 PAMP [175]. Thus, while additional research is needed to determine the role of endogenous expression of dUTPases in disease, the identification of novel properties of viral dUTPases should stimulate more research focused on whether these proteins could be exploited as novel molecular targets for the development of alternative dUTPase-based therapeutics.

## Figures and Tables

**Table 1:** Properties of human herpesviruses dUTPases.

Virus	Gene	Enzymatic Activity	Crystal Structure	Protein Homology ^b^ (%)	Required for In Vitro Replication	Immune Modulatory Function ^c^
HSV-1/2	UL50	Yes [20,21,22,23]	ND	29	No [13,24]	Induces IL-10, IL-12p70, IL-1β, IL-6, IL-8, TNFα in human PBMC and human dendritic cells (hDC) [7]
VZV	ORF8	Yes [14]	ND	24	No [14]	Induces IL-10, IL-12p70, IL-1β, IL-6, IL-8, TNFα in hPBMC and hDC [7]
HCMV	UL72	No [6]	ND	24	No [6]	ND
HHV-6A	U45	No [7]	ND	21	ND	Induces IL-10, IL-12p70, IL-1β, IL-6, IL-8, TNFα in hPBMC and hDC [7]
HHV-6B	U45	ND ^a^	ND	21	ND	ND
HHV-7	U45	ND	ND	23	ND	ND
EBV	BLLF3	Yes [23]	Yes [4]	100	ND	Induces IL-10, IL-12p70, IL-1β, IL-6, IL-8, IL-17A, TNFα in hPBMC and hDC [25,26,27,28,29] as well as IL-1β, IL-6 and IL-17 in vivo [30,31,32]
HHV-8	ORF54	Yes [33]	ND	31	ND	Induces IL-10, IL-12p70, IL-1β, IL-6, IL-8, TNFα in hPBMC and hDC [7] Downregulates NKp44L [34]

^a^ Not Done; ^b^ Maximum identity compared to EBV-dUTPase; ^c^ Independent of enzymatic activity.
